# Metabolomics, Microbiota, and In Vivo and In Vitro Biomarkers in Type 2 Severe Asthma: A Perspective Review

**DOI:** 10.3390/metabo11100647

**Published:** 2021-09-22

**Authors:** Cristiano Caruso, Stefania Colantuono, Alberto Nicoletti, Stefania Arasi, Davide Firinu, Antonio Gasbarrini, Angelo Coppola, Loreta Di Michele

**Affiliations:** 1 Allergy Unit, Fondazione Policlinico A. Gemelli, IRCCS, Catholic University of the Sacred Heart, 00100 Rome, Italy; stefania_colantuono@hotmail.it; 2 Digestive Disease Center, Medical and Surgical Sciences Department, Fondazione Policlinico Universitario Agostino Gemelli IRCCS, Catholic University of Sacred Heart, 00100 Rome, Italy; Antonio.gasbarrini@unicatt.it; 3 Internal Medicine, Gastroenterology and Hepatology, Fondazione Policlinico Universitario Agostino Gemelli IRCCS, Department of Internal Medicine, Catholic University of the Sacred Heart, 00100 Rome, Italy; alberto.nicoletti@guest.policlinicogemelli.it; 4 Area of Translational Research in Pediatric Specialities, Allergy Unit, Bambino Gesù Children’s Hospital, IRCCS, 00165 Rome, Italy; stefania.arasi@yahoo.it; 5 Department of Medical Sciences and Public Health, University of Cagliari, 09100 Cagliari, Italy; davidefirinu@yahoo.it; 6 Division of Respiratory Medicine, Ospedale San Filippo Neri-ASL Roma 1, 00100 Rome, Italy; coppolangelo@gmail.com; 7 UniCamillus, Saint Camillus International, University of Health Sciences, 00131 Rome, Italy; 8 Pulmonary Interstitial Diseases Unit, UOSD Interstiziopatie Polmonari Az Osp. S. Camillo-Forlanini, 00100 Rome, Italy; dottoressadimichele@gmail.com

**Keywords:** asthma, metabolomics, microbiota

## Abstract

Precision medicine refers to the tailoring of therapeutic strategies to the individual characteristics of each patient; thus, it could be a new approach for the management of severe asthma that considers individual variability in genes, environmental exposure, and lifestyle. Precision medicine would also assist physicians in choosing the right treatment, the best timing of administration, consequently trying to maximize drug efficacy, and, possibly, reducing adverse events. Metabolomics is the systematic study of low molecular weight (bio)chemicals in a given biological system and offers a powerful approach to biomarker discovery and elucidating disease mechanisms. In this point of view, metabolomics could play a key role in targeting precision medicine.

## 1. Introduction

Asthma is a chronic respiratory disease characterized by shortness of breath, airway inflammation, and airway hyperresponsiveness. It is a heterogeneous, multifaceted disease with variable severity and treatment response. For patients with severe asthma who experience poor symptom control and/or frequent asthma exacerbations, the addition of biological therapies to controller medication is becoming the new standard of care [1]. The introduction of novel treatments with biologics targeting type 2 inflammation pathways urges the development of clinical decision-making tools to guide therapy based on underlying asthma endotypes driving the disease in an individual patient. Severe asthma is driven by heterogeneous molecular mechanisms. Nevertheless, the response rates reported in phase III regulatory trials make it highly desirable to identify biomarkers able to predict response to these biological treatments in severe asthma patients [2]. The omic approach could represent a useful and very interesting tool to better phenotype severe asthma patients and then target biological therapy. However, multi-omics projects in asthma are challenging in terms of costs and require computational and human resources. Therefore, their success requires the coordination and collaboration of diverse research groups from different disciplines in an international multicenter approach. Due to the demanding nature, multi-omics projects in asthma are scarce but have proven value in the comprehensive evaluation of molecular processes in asthma pathogenesis [3]. Metabolomics is the systematic study of low molecular weight (bio)chemicals in a given biological system [4] and offers a powerful approach to biomarker discovery and elucidating disease mechanisms; inter- and intra-study irreproducibility often arise from technical inconsistency, absent metabolite inclusion criteria, inappropriate or overtrained chemometric analyses and poor reporting standards [5].

## 2. Discussion

### Perspective Approach in Metabolomics in Severe Asthma Research

In this review, we described asthma omics fields (genomics, epigenomics, transcriptomics, metabolomics, and proteomics) and how these have contributed to our current understanding, for example, of childhood asthma endotypes, taking into account challenges and opportunities of the different approaches. Commonly used approaches are currently limited by the three I’s: integration, interpretation, and insights. Post integration, these large data sets aim to yield views of cellular systems at high resolution for transformative insights into processes, events, and diseases through various computational and informatics frameworks [6]. Despite the large amount of data the new omics era has provided on type 2 high- and type 2 low-driven patterns of airway inflammation, the only biomarkers that are currently recommended in the The Global Initiative for Asthma (GINA) guidelines, as well as a recent ERS/ATS task force on the management of severe asthma to guide treatment with biologics, are total and/or specific serum IgE, blood eosinophils, and FeNO. To apply the latest omics technology to guide treatment in severe asthmatic, there is a need for biomarker discovery studies on biologics in children, validation studies, biomarker-guided studies, and implementation studies. So far, most published biomarker studies are in the phase of biomarker discovery for asthma prediction, asthma severity, or corticosteroid response. These studies often report associations. Validation studies should include functional validation of biomarkers using ex vivo models, development of targeted panels of selected omics markers preferably incorporated in a simple clinical test or treatment algorithm, and clinical validation in independent cohorts. An increasing number of studies reporting on microbiome association with asthma leaves us with a promising tool that in the near future may allow implementing a microbiomic phenotype as a tool for the prediction of disease progression or responses to medications [7].

## 3. Methods

### 3.1. Strategies in Multi-Omics Research

The local and systemic responses are highly activated in asthma, and multiplexed analysis indicates a broad range of inflammatory mediators and responses that are better understood but still largely unsolved [6].

In the last decade, the understanding of the immune response of asthma and the integration of systems biology approaches (genomics, methylomics, transcriptomics, proteomics, metabolomics, etc.) has led to the identification of disease clusters and asthma endotypes [7].

Disease clusters are potentially linked to disease markers and tailored treatment targets by pathobiological mechanisms.

Metabolomics systematically studies the variety of endogenous metabolites (e.g., small molecules as amino acids, carbohydrates, lipids, nucleotides, and organic acids) in biological specimens (e.g., blood, serum, exhaled breath and urine, cells, and tissue) and represents a comprehensive assessment of ongoing biological processes in states of health and disease. The main biological pathways that can be mapped by metabolomics are an immune response, inflammation, and signaling; metabolism of amino acids, sugars, bile acids, steroids, and lipids; oxidative stress, redox balance, and hypoxia; energy homeostasis; and DNA methylation [8].

The heightened immune response is linked to a shift in tissue metabolism as a result of inflammation-driven recruitment of inflammatory cells, such as neutrophils and monocytes. At sites of inflammation, there are metabolic changes due to an increased nutrient, energy, and oxygen demand to accomplish cell migration, phagocytosis, and other processes [9]. In addition to altered cellular metabolism, extracellular pathways can generate biologically active molecules capable of initiating and modulating inflammatory responses. There is a growing body of evidence that microbiota plays a pivotal role in the modulation of amino acid and lipid metabolism, which exert a strong effect on the immune system [10].

Understanding the metabolic implications of chronic inflammatory processes is, therefore, a crucial need.

Two analytical approaches are employed in metabolomics, untargeted and targeted. Untargeted metabolomics measures a broad range of metabolites in a biological sample, giving an advantage for the investigation of the complex interaction between metabolites from multiple pathways in a holistic, hypothesis-free analysis of (potentially) all the metabolites present in the analyzed sample [11].

The untargeted approach also allows the discovery of novel metabolites; however, it produces a bulk of data that is difficult to analyze. Untargeted approaches require protocols for unbiased data coverage, validation, and quality control of the obtained results and resources to characterize newly identified metabolites.

Targeted metabolomics is based on the focused quantification of predefined known groups of metabolites. This method has several advantages over untargeted metabolomics, including higher sensitivity and selectivity as the metabolites captured are based on previous experiments and libraries of specific metabolites, enzymes and kinetics, and biochemical pathways. In targeted metabolomics, optimized protocols for sample preparation can decrease the number of high-abundance molecules that may interfere, and novel associations between metabolites can be analyzed in the context of a specific illness.

To generate a metabolomic profile, spectroscopic and spectrometric techniques are used, such as high-field nuclear magnetic resonance spectroscopy (NMR) and mass spectrometry (MS), and separation techniques coupled to mass spectrometric detection, such as High-performance liquid chromatography (HPLC) , ultra-HPLC (UHPLC), gas chromatography (GC), capillary electrophoresis (CE), 2D chromatography, and supercritical fluid chromatography. The matrix of the sample, the concentration and properties of the metabolites, and the quantity of sample drive the choice of a given method [4].

NMR is reproducible and non-destructive for the molecules, can be automatized, results in fast analyses, and provides the opportunity to simultaneously measure many types of small molecules from the metabolome, with detection limits in the order of μM or nM [4]. A drawback is that NMR has poor sensitivity as compared to MS, and the NMR spectra can produce overlapping signals among metabolites rendering quantification unfeasible with low-abundant metabolites. MS is based on the separation of molecules of a biofluid (or other biological sample) and their fragments by their mass-to-charge ratio. This technique has a higher sensitivity, better reproducibility, and selectivity than NMR. LC-MS and GC-MS platforms require minimal sampling compared to the wide collection of metabolic fluctuations that can be assessed. The main drawbacks include the costs to run a laboratory compared to NMR and the reduced levels of fragmentation in LC-derived ionization [4].

In contrast to NMR, the detectability of a metabolite in MS depends on the ionization efficiency that is influenced by the sample composition and by analyte separation before the mass analyzer. The coupling of different platforms with different ionization and/or chromatographic strategies has been recently applied to detect untargeted metabolomic signatures in food allergy and asthma [4,12].

In the last couple of years, the challenge of personalized medicine entered the field of respiratory medicine and asthma and drove the first identification of endotypes, each linked to different biological mechanisms that have been partially elucidated in their inherent complexity [13].

### 3.2. Metabolomics in Type 2 Inflammation

The observed levels of metabolites result from the dynamic changes in the genome, transcriptome, and proteome. In addition, they reflect the current phenotype of a particular organism. Therefore, it is considered that the metabolome is related to the genotype-to-phenotype gap [14]. Some metabolomic studies explored putative biomarkers in Th2-driven diseases such as chronic rhinosinusitis, nasal polyposis, atopic dermatitis, and food allergy.

### 3.3. Asthma

In the last decade, an increasing interest in metabolomics research applied to asthma has been observed. Metabolomics profiling of asthmatic patients could serve both as a diagnostic tool and as a biomarker. Currently, there is a limited number of studies concerning the discrimination of asthmatic patients from healthy controls using liquid or gas chromatography-mass spectrometry (LC-MS or GC-MS) and nuclear magnetic resonance (NMR)-based metabolomics in several biofluids such as serum, urine, and volatile organic compounds (VOCs) in exhaled breath and exhaled breath condensate (EBC) [15].

In general, exhaled VOCs provide a composite biomarker signal based on pattern recognition profiles that seem correlated with eosinophils both in blood and in alveolar fluid [16].

According to the same concept of exhaled VOCs, eNose may be used as a predictor of poor asthma control [17], showing higher sensitivity than FeNO or sputum eosinophilia in the prediction of clinical efficacy of systemic corticosteroids. However, further validation studies are needed; therefore, this method cannot be considered at the time of clinical use.

Recently, several urinary metabolites have been detected as possible biomarkers in asthma, considering that they are noninvasive, easily assessed, and allow multiple analyses. Urinary leukotriene E4 (LTE4), prostaglandins D2 (PGD2), and bromotyrosine, for example, are currently investigated, but their role as biomarkers is still limited to research studies and requires further evaluation [18].

Among metabolites, fatty acids play an essential role in the development and resolution of inflammatory pathways relevant to the pathophysiology of asthma; therefore, they represent an interesting class of mediators under investigation in this field. Some lipid mediators promote inflammation, whereas others are involved in the resolution phase, even if it has been documented that the same mediator might be proinflammatory in one disease or tissue and it can be anti-inflammatory in another. Most of the lipid mediators that regulate inflammation are metabolites derived from omega-6 (n-6) or omega-3 (n-3) fatty acids, including arachidonic acid (AA; 20:4n-6), linoleic acid (LA; 18:2n-6), eicosapentaenoic acid (EPA; 20:5n-3), and docosahexaenoic acid (DHA; 22:6n-3), as is the case for the AA-derived prostaglandin (PG) E2. The main AA-derived mediators of inflammation in asthma are PGs and cysteinyl leukotrienes (CysLTs). There are many other eicosanoids that have been implicated; however, their roles remain somewhat controversial compared with PGs and leukotrienes (LTs) [19].

In this scenario, metabolomics appears to be a promising strategy to increase our phenotyping and endotyping abilities of asthmatic patients. Considering this also changes in diet have the potential role in modifying the anti-inflammatory/proinflammatory balance, and this can allow us to deepen new intervention strategies.

#### 3.3.1. Chronic Rhinosinusitis (CRS)

Based on metabolomics studies, the metabolic fingerprinting strategy, metabolic profiling, and quantitative targeted metabolomics are identified as useful in CRS development.

Multiple clinical studies have reported dysregulated fatty acid metabolism in severe asthma and aspirin-exacerbated respiratory diseases. Miyata et al. [20] focused on the metabolic regulation of cysLTs in inflammatory eosinophils, a major cellular source of these mediators, and explained how fatty acid metabolism of eosinophils could be regulated in asthma and aspirin-exacerbated respiratory diseases (AERD), which are often complicated with eosinophilic rhinosinusitis. Further integrated analysis identified type 2 cytokines or microbial components as inducers of fatty acid metabolic abnormality, suggesting the importance of the tissue environment of nasal polyps to affect cellular inflammatory characteristics. Recent clinical trials reported the usefulness of antibody drugs for type 2 cytokines to improve disease outcomes of chronic eosinophilic rhinosinusitis. [21].

Fazlollahi et al. [22] demonstrated the application of an untargeted lipidomic study for the analysis of lipids, among other metabolites. The levels of fatty acids were, on average, 10-fold higher in CRS patients with concomitant NP growth compared to other groups, which suggested an altered lipid metabolism in the sinus mucosa samples. Cholesteryl palmitoleate was present in CRS patients with concomitant NP, whereas in the case of those with CRS without nasal polyps (CRSsNPs), it was not detectable; cholesteryl arachidonate was present in all CRS sinus mucosa samples, but not in NP.

In the study conducted by Miyata et al. [23], a multi-omic (transcriptomics, proteomics, and metabolomics) approach was proposed for the isolation of CD69hi CCR3low CXCR4-siglec-8int eosinophils from NP from patients with eosinophilic rhinosinusitis compared to healthy controls, observing significantly lower amounts of both cyclooxygenase (COX) and lipoxygenase (LOX) products in the NP group compared to the second. However, no significant changes were noted in the level of arachidonic acid, a precursor of COX and LOX, which suggests that patients with NP-EOS may display unique lipid mediator profiles that can also affect specific EOS phenotypes.

In a targeted metabolomic study, Tsybikov et al. [24] used the immunoassay method for the determination of neopterin, a product of human monocytes and macrophages that is an indicator of Th1-polarized immune activation. The levels of neopterin were significantly elevated in CRSsNP patients compared to healthy controls in both sera and nasal secretion samples. Furthermore, neopterin levels measured in nasal secretions were also significantly elevated in CRSsNP patients compared to those with CRSwNP. Therefore, this metabolite would be proposed as a potential biomarker only for CRSsNP patients.

Broza et al. [25] analyzed volatile organic compounds (VOCs) emitted from body sources. However, to assess the potential role of nNO as a diagnostic and monitoring marker, the studies should be validated in a larger group of both CRS patients and healthy controls.

#### 3.3.2. Food Allergy and Atopic Dermatitis

Metabolomics studies of atopic children focused on the metabolism of different pathways, including tyrosine and tryptophan, lipids, Polyunsaturated fatty acids (PUFAs), Short-chain fatty acids (SCFAs), and bile acids, to investigate the potential role of these metabolites for discriminating between health and atopic diseases and for identifying different endotypes of atopic disease.

The pattern of SCFAs is altered as the maturation of the gut microbiome takes place until the age of approximately 3 to 4 years [26]. Assfalg et al. [27] investigated urine samples from infants with AD, and their analysis indicated increased 2-hydroxyburyrate in infants with asthmatic disease compared to healthy control.

Huang et al. [28] found higher levels of tryptophan and indolelactic acid in children with AD with elevated specific-IgE compared to those with normal IgE and HCs, respectively, while these metabolites did not differ among children with AD and normal IgE levels compared to HCs.

They also investigated targeted eicosanoids in serum of children with AD and showed different levels in children with AD with high IgE and normal IgE levels, respectively, compared to HC. Hydroxyl octadecadienoic acids (HODEs) and most of the investigated hydroxy eicosatetraenoic acids (HETEs) were significantly higher in the two AD groups compared to HCs. Children with AD and high IgE expressed significantly lower levels of 8, 9, 11, 12, 16, 19, 20-HETEs, and 9, 13-HODEs than those with normal IgE. Moreover, the levels of PGE2 and LTB4 were significantly higher in the AD groups. However, PGE2 and LTB4 could not discriminate between AD groups with high IgE levels from normal IgE levels.

In the same study, glycocholic acid and three primary conjugated bile acids: taurocholic acid, taurochenodeoxycholic acid, and glycochenodeoxycholic acid, were significantly decreased in the serum of children with AD independently of high or normal IgE levels compared to HC. On the other hand, children with AD and high IgE had elevated levels of two unconjugated bile acids: cholic acid and chenodeoxycholic acid, compared to HCs. Moreover, they demonstrated significantly altered levels of three sphingomyelins in children with AD with elevated IgE compared to HCs.

Crestani et al. and another most recent study [10,29] identified that 81 metabolites were altered in children with food allergies compared to children with asthma. The metabolite 5-hydroxyindoleacetate in the tryptophan metabolism and the five metabolites: 3-(4-hydroxyphenyl) lactate, 3-methoxytyramine sulfate, 4-methoxyphenol sulfate, phenol glucuronide, and phenol sulfate in the tyrosine metabolism, were significantly different in children with asthma compared to children with food allergy. The authors concluded that children with a combination of asthma and food allergy capture a metabolomic signature concordant with food allergy alone rather than asthma. Pathway analysis revealed that one of the most dysregulated pathways associated with the presence of food allergy compared with asthma included secondary bile acid metabolism.

They showed that sphingolipid metabolism was affected by food allergies. Sphingolipid metabolism involves the conversion of ceramide into sphingomyelins and conjugated ceramides. The transformation of ceramides to sphingomyelin and acylceramide and glucosylceramide was decreased in children with food allergies compared to non-atopic controls and children with asthma (*p* < 0.05). Moreover, several lysophospholipids discriminated children with food allergies from asthmatic children and food allergies from non-atopic controls. Lower levels of phospholipids were associated with food allergy with or without concurrent asthma (all *p* < 0.05). The pathways strongly altered with the presence of food allergy included dihydrosphingomyelins, lactosylceramides, sphingomyelins, and hexosylceramides, among others.

### 3.4. Biomarker Research in Type 2 Severe Asthma

The hallmarks of asthma are represented by chronic airway inflammation, variable airway obstruction, airway hyperresponsiveness (AHR), and structural changes within the lower airways, generally associated with clinical symptoms [20].

Although most patients with asthma can be effectively treated with currently available medications, it is estimated that half of them have uncontrolled disease, mostly due to poor treatment adherence, misuse of inhaler delivery devices, persistent exposure to triggers, and/or poorly managed comorbidities [20].

Severe asthma (SA) is a relevant challenge not only for patients and physicians but also for society since the health care cost for SA constitutes more than 50% of that for asthma [21].

Over the years, clinicians have identified several phenotypes of SA, based on the presentation, the age of onset of symptoms, the severity of the disease, and the presence of other conditions such as allergic status and/or eosinophilia, with different long-term outcomes, rate of exacerbations and response to therapy with corticosteroids [22].

Integration of genetics, biology, and clinical features and a better understanding of asthma pathogenesis led to the identification of two major endotypes based on the inflammatory pathway involved, that is, type 2 high (T2-high) and type 2 low (T2-low).

Patients with T2 high asthma account for approximately 60% of patients with SA. They are often responsive to inhaled corticosteroids, and their disease is associated with an increased expression of T2 cytokines such as interleukin IL-4, IL-5, and IL-13 driven either by allergic mechanisms through the Th2 lymphocytes or by other exposures that activate the innate immune system through the type 2 innate lymphocytes (ILC-2 cells). Several subtypes of T2 high asthma have been identified, including early onset allergic asthma, late-onset eosinophilic, and aspirin-exacerbated respiratory disease (AERD) [30,31,32].

T2 low (non-T2) asthma, although very heterogeneous, is usually characterized by the presence of neutrophilic or minimal (pauci-granulocytic) airway inflammation. Two postulated mechanisms leading to neutrophilic inflammation include dysregulated innate immune response, with possible neutrophilic intrinsic abnormalities and activation of the Th17 inflammatory pathway. Subtypes of T2 low or non-T2 asthma include those with neutrophilic asthma associated with smoke exposure, elderly asthma, and late-onset obese asthma [33].

In the era of personalized or precision medicine, also for asthma, it is extremely important to phenotype the condition in an unbiased way and to define some biomarkers able to predict above all the course of the disease and the response to therapy [34].

To date, there are multiple phenotypes that can be partially differentiated by measuring the specific characteristics of the disease, but the pathogenetic mechanisms are complex, and it is still a challenge to choose suitable biomarkers to adequately stratify patients, especially after the introduction of biologicals in asthma treatment. According to the U.S. National Institute of Health (NIH) biomarker working group, a biomarker is defined as “a characteristic that is objectively measured and evaluated as an indicator of normal biologic processes, pathogenic processes, or pharmacologic responses to a therapeutic intervention.” [35].

Useful biomarkers in respiratory disease can be obtained by several different types of clinical samples, such as bronchoscopic samples, induced sputum, blood, urine, and exhaled gases with several potential applications in the management of SA such as understanding biology, ameliorating diagnosis, screening, assessment of severity, control, or prognosis of patients and in the identification of endotypes. Moreover, there is a growing application in clinical trials and safety monitoring by identifying pharmacodynamic or predictive-of-response biomarkers, and a variety of response outcomes is used in the different clinical trials, as well as a huge range of potential predicting factors.

Principal biomarkers in T2 asthma and their common site of sampling are summarized in Table 1.

Eosinophilia, both in blood and in sputum, is likely to be one of the most studied and used biomarkers in T2 severe asthma.

Sputum eosinophil count is considered a useful tool to assess eosinophilic airway inflammation and may provide important information regarding the eosinophilic asthma phenotype, prognosis, and potential response to ICS therapy [36]. Increased sputum eosinophil counts are associated with an elevated risk of exacerbations [37], and a sputum eosinophil count of ≥ 2% is suggestive of underlying eosinophilic airway inflammation; however, despite its potential benefits for the assessment of asthma phenotype, it is routinely performed only in specialized centers. Another interesting biomarker of eosinophilic inflammation in asthmatic patients is the eosinophil cationic protein (ECP), a cytotoxic protein stored in granules and released by activated eosinophils in response to many different stimuli in reason of its toxic effects on bacteria, parasites, viruses, and epithelial cells in the airways. ECP levels can be assessed in several samples, most commonly serum or sputum. In serum, it has been observed an increase in ECP levels among asthmatics compared to healthy non-smoking subjects, but other respiratory diseases such as chronic obstructive pulmonary disease (COPD), interstitial lung diseases, acute respiratory infections, eosinophilic granulomatosis with polyangiitis (EGPA), chronic eosinophilic pneumonia and allergic rhinitis may be associated with a similar increase. On the other side, ECP levels have been reported to decrease in response to treatment with the anti-IL-5Ra drug benralizumab [38]. To date, ECP has shown prognostic properties associated with increased risk of exacerbations and uncontrolled asthma; however, its predictive properties are still limited, principally due to overlap with many other pulmonary disorders. Similar to the ECP, eosinophil-derived neurotoxin (EDN) is another granule protein released by activated eosinophils with a growing body of evidence for its utility as a biomarker in asthma. EDN can be sampled from different body fluids such as serum and urine and showed an association with eosinophilic airway inflammation, disease severity, and exacerbation risk [39].

Recently, also eosinophil peroxide (EPX), a major granule released by activated eosinophils, is under investigation as a possible biomarker in T2 asthma because its level has been correlated with sputum eosinophils in patients with poorly controlled eosinophilic asthma, allergic and other eosinophilic diseases. EPX has been assessed in nasal and pharyngeal swabs, but a novel point-of-care method for rapid quantification of eosinophil peroxidase in sputum has recently been proposed to identify patients with airway eosinophilic inflammation. EPX levels decreased in patients treated with anti-eosinophilic biologics, and also, for this reason, it can be an interesting biomarker in the management of T2 asthma; however, it is not still available for clinical use. Among serum biomarkers in T2 asthma, an important role is played by immunoglobulin E (IgE), which is a pivotal mediator in allergic asthma phenotype. It binds to IgE receptors on mast cells and basophils, producing cytokines and causing the release of several mediators of T2 inflammation. Serum IgE closely correlates with the risk of asthma, and increased IgE levels are connected with the presence of wheezing and with the severity of disease both in adults and children as an independent factor associated with the presence of sputum eosinophilic inflammation [39].

From a clinical point of view, total IgE levels are commonly used in the assessment of allergic asthma and in the identification of patients suitable for biological therapy with omalizumab, even if IgE levels cannot be used as a predictive biomarker to determine response to this treatment.

Periostin is a matricellular protein that interacts with multiple signaling cascades and cellular interactions within the extracellular matrix involved in airway remodeling and inflammation. Bronchial epithelial cells are able to produce periostin in response to IL-13, and for this reason, it has been proposed as a possible predictive and prognostic biomarker in T2 asthma as a surrogate of IL-13 activity or more in general of T2 inflammation. The utility of serum periostin was investigated in several studies; however, its levels may fluctuate with age, especially in growing children and in other respiratory diseases; thus, its clinical use remains uncertain [26].

It has also been reported the possible role of dipeptidyl peptidase-4 (DPP-4) as a candidate predictive biomarker for the response to anti-IL-13 treatment, even if further studies are needed to confirm the potential role of DPP-4 as a surrogate T2 biomarker. Several novel noninvasive methods have been assessed in the last decades to investigate new possible biomarkers in asthma, and multiple inflammatory components have been evaluated for their potential, including fractional exhaled nitric oxide (FeNO) and volatile organic compounds (VOCs) in exhaled breath and exhaled breath condensate (EBC). FeNO represents a reproducible, noninvasive, and easily measurable indicator of IL-13-driven and corticosteroid-responsive airway inflammation due to IL-13 activation of inducible nitric oxide synthase, which increases the production of FeNO [36].

According to the ATS recommendations, FeNO > 50 ppb (adults) and > 35 ppb (children) are indicative of eosinophilic inflammation, while eosinophilic inflammation is unlikely for FeNO < 25 ppb (adults) and < 20 ppb (children), nevertheless FeNO level may be affected by several confounders, including demographics, smoking, atopy, and diet [37].

From this panoramic view, it seems evident that it is unlikely that a single biomarker can reflect the complex pathophysiology and heterogeneity in asthma. The right combination of various biomarkers in different phenotypes is under investigation and may allow clinicians to better phenotype and evaluate the patient toward an individual approach and personalized medicine.

### 3.5. Microbiota in Severe Asthma

The study of microbiota has revolutionized the understanding of human diseases. Indeed, the advent of metagenomics techniques has revealed the presence of microorganisms in sites that were previously considered sterile, such as the lower respiratory tract (LRT) [38]. The microbiota of the respiratory tract consists of bacteria, fungi, and viruses. These microorganisms establish a complex interaction with the immune system and play a role in the development of some immune-mediated disorders. The anatomical and physiological features of the airways influence their colonization: the qualitative and quantitative composition of the microbiota in each site is the result of the dynamic balance between migration and elimination of microorganisms with the airflow, the growth rates of microbes, and the clearance by the immune system [39]. For these reasons, microbial density is higher in the upper respiratory tract (URT). Furthermore, from birth, several environmental factors, such as the birth mode, feeding type, vaccinations, diet, infections, antibiotic use, exposure to smoke, and other detrimental substances, affect the composition of the microbiota [40]. Biodiversity, microbial balance, and the relative abundance of keystone species are associated with beneficial functions and health [41]. Moreover, some microorganisms, known as keystone species, are crucial to maintain the structure of the microbial community and exert beneficial effects, favoring the integrity of the barrier, enhancing the immune system, and directly contrasting the growth of pathogens. On the other hand, the imbalance of the microbial niche, known as dysbiosis, is a potential driver for the development of diseases [42].

Several studies have demonstrated that Proteobacteria are the most abundant phylum in patients with asthma compared with controls [43]. This phylum includes potentially pathogenic bacteria that belong to *Moraxella*, *Haemophilus*, and *Neisseria genera*. Interestingly, specific inflammatory phenotypes in severe asthma have shown relevant differences in the composition of the respiratory microbiota. In particular, a significantly reduced microbial diversity and evenness characterize the microbiota of patients with neutrophilic phenotypes. Moreover, a high abundance of *Haemophilus* and *Moraxella genera* has been observed in this population [44]. This may be the result of both an overgrowth of pathogens and a decrease in *Gemella*, *Streptococcus,* and *Porphyromonas genera*, which appear to be particularly sensitive to this particular inflammatory environment [45,46]. The microbiota of patients with atopic asthma, which is usually associated with an eosinophilic inflammatory phenotype, is characterized by an increased abundance of the *Haemophilus*, *Fusobacterium*, *Neisseria*, *Porphyromonas genera,* and the *Sphingomonodaceae* family, and a decrease in *Mogibacteriaceae* family and *Lactobacillales* order [47].

The study of the microbiota may provide crucial implications for the therapeutic management of the disease. For example, the presence of *Actinobacteria phylum* correlates with the expression of FKBP5, a molecular marker of response to steroids [48]. In a population of patients with atopic asthma, non-responders to inhaled corticosteroids presented enrichment in *Microbacteriaceae* and *Pasteurellaceae* families before the start of the treatment, whereas the responders had an increased relative abundance of *Streptococcaceae*, *Fusobacteriaceae*, and *Sphingomonodaceae* families. On the other hand, the use of both systemic and inhaled corticosteroids is associated with a perturbation in the composition of the microbiota with enrichment of *Proteobacteria* and *Pseudomonas* and a decreased abundance of *Bacteroidetes*, *Fusobacteria*, and *Prevotella* [49].

Insights from the interaction between pharmacological treatments, the responsiveness of the immune system, and the microbiota may help to better target pharmacological treatments. Hence, further studies are needed to better understand the influence of pharmacological treatments in such a complex balance.

### 3.6. Sleep-Related Breathing Disorders and Omics in Severe Asthma

The third International Classification of Sleep Disorders (ICSD-3) identifies seven major categories of sleep disorders that include insomnia disorders, sleep-related breathing disorders, central disorders of hypersomnolence, circadian rhythm sleep-wake disorders, sleep-related movement disorders, parasomnias, and other sleep disorders.

Within respiratory sleep disorders, obstructive sleep apnea (OSA) is the most studied and documented one in the asthmatic patient and, in particular, in severe asthmatics. Both asthma and OSA are common diseases that may easily coexist; however, the prevalence of OSA in asthmatic patients is up to three times higher than in non-asthmatics [50,51,52].

The overlap between asthma and OSA has particular peculiarities, and already in 2013, it was defined as an alternative overlap syndrome to distinguish it from overlapping with COPD [53]. Once OSA is established, the intermittent hypoxemia linked to respiratory events causes oxidative stress and an increase in IL-6, CRP, and TNF, leading to neutrophilic inflammation. In asthmatic patients, persistent OSA can cause a shift toward a Th1 type of inflammation, complicating the evolution of the disease and the response to therapy [54].

Recently, -omics sciences are also applied in OSA patients. The first proteomic approach in OSA dates back to 2018 with the study by Zhang [55], which highlighted how intermittent hypoxemia is associated with the increase in 4 proteins (CRP, haptoglobin, fibronectin, platelet factor 4) released into the extracellular fluid from part of microvesicles of cells subjected to hypoxic insult. Since then, proteomics and metabolomics studies in OSA have intensified, highlighting several molecules that may represent biomarkers of OSA. Hypoxemia selects the microbial flora and modifies intestinal permeability. The bacteria, thus selected, release toxic metabolites such as trimethylamine (TMA) and trimethylamine oxide (TMAO), which can be considered as biomarkers for predicting cardio/vascular damage in patients with OSA [56].

We can therefore hypothesize that the patient with asthma and obstructive sleep apnea overlap syndrome presents alterations of the intestinal microbiota secondary in part to intermittent hypoxia, in part to the fragmentation of sleep that derives from the condition of severe asthmatic that in itself acts with another mechanism in altering the intestinal microbiota and the presence of OSA may alter the biomarker profile of these patients. To our knowledge, there are no studies that have observed whether the dual mechanism of alteration of the microbiota can lead to even more unfavorable outcomes.

It appears necessary to investigate the possible presence of sleep disorders in all subjects with uncontrolled asthma and especially in severe asthma. OSA should always be treated as soon as possible in order to avoid inflammation switching. This could justify the partial lack of therapeutic response to the biological drugs currently available for severe asthmatics who are in a situation of transition from Th2 to Th1 inflammation. Metabolomics and proteomics studies could have decisive implications in identifying biomarkers of this inflammatory overlapping situation.

### 3.7. Prevention of Asthma at Young Age, Findings from Metabolomics in Pediatric Asthma

Metabolomics represents an appealing approach throughout the lifecycle in asthma research. Specifically, in the context of pediatric asthma research, monitoring environment-gene interactions by measuring metabolites has several potential peculiar applications [57]. Furthermore, it can potentially help in better elucidating the underlying mechanistic pathways and, therefore, discriminate among different asthma phenotypes that will support in tailoring personalized medical treatment and provide better symptom care in pediatric asthmatic patients and preventive measures to avoid the development of asthma disease.

However, evaluating early life respiratory morbidity in infants is quite challenging. In vivo experiments with environmental exposures (including viral infection) are difficult to conduct in infants due to ethical restrictions [58]. Moreover, results from investigations conducted in adults may not be simply translated to infants and children because of differences in immunological systems, pulmonary development, and heterogeneous responses to environmental exposures based on age.

The first pediatric asthma metabolomics report dates back to less than two decades ago [59]. The authors applied untargeted nuclear magnetic resonance (NMR) metabolomics on exhaled breath condensate (EBC) samples of 25 asthmatic patients and 11 healthy controls aged between 7 and 15 years. The combination of exhaled nitric oxide and FEV-1 discriminated children with asthma and healthy children with a success rate of approximately 81%, whereas selected signals from NMR spectra identifying acetylated and oxidized compounds offered even slightly better discrimination (approximately 86%) [59].

From then on, further studies have been performed to distinguish between asthmatic and healthy children, but also to identify specific phenotypes of asthma severity [60,61] to differentiate wheezers from non-wheezers [62], transient wheezers from asthmatic patients [63], and controlled from uncontrolled asthma [64]. To these aims, several techniques on different types of samples (e.g., exhaled breath, exhaled breath condensate, plasma, serum, urine, amniotic fluid) have been applied, including the following: targeted liquid chromatography-mass spectrometry (targeting adenosine monophosphate and purine and leukotriene [65]); untargeted liquid chromatography-mass spectrometry; and targeted gas chromatography-mass spectrometry (targeting volatile organic compounds and alkanes, alkenes, aldehydes, and ketones) in exhaled breath and exhaled breath condensate samples [66] but also in others.

A combined approach integrating metabolomics to genetics has shown that sphingolipid metabolism and immune response pathways are associated with asthma control. Specific metabolic pathways (i.e., glycine, serine, and threonine metabolism and *N*-acylethanolamine and *N*-acyltransferase pathways) have been associated with specific patterns of asthma severity in children by applying untargeted LC-MS in plasma samples [67] and targeted NMR and liquid chromatography-mass spectrometry to urine samples [66,68]. Specific urine metabolic profiles measured at four weeks through untargeted liquid chromatography-mass spectrometry were related to asthma development before six years of age, differing from healthy children. Urine metabolites may be useful not only as biomarkers of asthma disease versus healthy states [69] but also as predictors of the progression of the disease [70].

Another field of application of metabolomics in pediatric asthma is overweight asthma patients. One study performed in children with obese-asthma phenotype found that overweight/obese children had lower FeNO measurements and supranormal spirometric parameters than normal-weight asthmatic children [71]. These respiratory features can be explained by dysanapsis or incongruence in the growth of airways and lung parenchyma. With respect to low FeNO values in overweight/obese asthmatic children, increased adiposity could lead to neutrophilic airway inflammation rather than eosinophilic, and FeNO is a surrogate marker for eosinophilic airway inflammation. Clinical implications are relevant since current asthma pharmacotherapy is based on treating eosinophilic airway inflammation and not for neutrophilic. As a result, obese asthma in pediatric cases requires alternatives in therapeutic management.

Overall, the metabolomics approach could be a noninvasive, promising technique to identify reliable candidate markers to stratify pediatric asthmatic patients by asthma phenotypes and plausible therapeutic targets. Further well-conducted studies are awaited.

## 4. Conclusions

Metabolomics has already been successfully applied in various fields of science, showing promise in maintaining safety and efficacy in clinical real-life research. Moreover, the latest developments and advances, limitations, future evolution, and applications of metabolomics are also critically discussed, providing valuable insights for further research directions in severe asthma biomarkers analysis. Corresponding to the fast development of analysis techniques, metabolomics has achieved great progress with exciting findings linking to biological systems and biomarkers analysis, in which it is possible to analyze more than 1000 metabolites in a single run or using an integration of various analytical methods, showing very great potential. However, for untargeted metabolomics, several procedures should be further refined to control analytical data quality and develop standardization of protocols for meaningful, accurate, and precise management of untargeted studies in biomarker analysis research.

## Figures and Tables

**Table 1:** Principal biomarkers in T2 asthma and their common site of sampling in clinical use.

Site of Sampling	Biomarker
Peripheral blood	Eosinophils
	ECP
	EDN
	IgE
	Periostin
	DPP-4
Sputum	Eosinophils
	EPX
Exhaled breath	FeNO

Abbreviations: eosinophil cationic protein (ECP), eosinophil-derived neurotoxin (EDN), immunoglobulin E (IgE), dipeptidyl peptidase-4 (DPP-4), eosinophil peroxide (EPX), fractional exhaled nitric oxide (FeNO).

## Data Availability

Data is contained within the article.
